# Supplementary material for: Adaptive model selection in photonic reservoir computing by reinforcement learning
Source: Sci Rep. 2020 Jun 22;10:10062. doi: 10.1038/s41598-020-66441-8 (PMC7308406; doi:10.1038/s41598-020-66441-8)
Supplement: Supplementary file 1 — Supplementary information. [file 41598_2020_66441_MOESM1_ESM.pdf]

Supplementary information:
Adaptive model selection in photonic reservoir computing by reinforcement learning

Kazutaka Kanno[1,*], **Makoto Naruse**[2], **and Atsushi Uchida**[1]

[1]Department of Information and Computer Sciences, Saitama University 255 Shimo-Okubo, Sakura-ku, Saitama City, Saitama, 338–8570 Japan

[2]Department of Information Physics and Computing, Graduate School of Information Science and Technology, University of Tokyo, 7-3-1 Hongo, Bunkyo-ku, Tokyo, 113-8654 Japan

*kkanno@mail.saitama-u.ac.jp

Results of Dependence of Model Selection on Randomly switching

We demonstrated the scheme for periodic switching of the model selection in the main text. The switching of the model selection may randomly occur in general situations. Thus, we change the duration of the switching of the model selection and investigate the transient time for which the correct model selection rate (CMSR) reaches 1, after the model switching occurs. Figure S1 shows the temporal evolution of the correct model selection rate for different switching times. In Fig. S1(a), the first 100 data points is generated from the Lorenz model, and the model is switched to the Rössler model at $n = 100$. Conversely, the model is switched at $n = 400$ in Fig. S1(b). The correct model selection rate reaches 1 in both case, however, the transient time required for CMSR = 1 in Fig. S1(b) is longer than that in Fig. S1(a). This result indicates that the performance of the model selection decreases for a longer n.

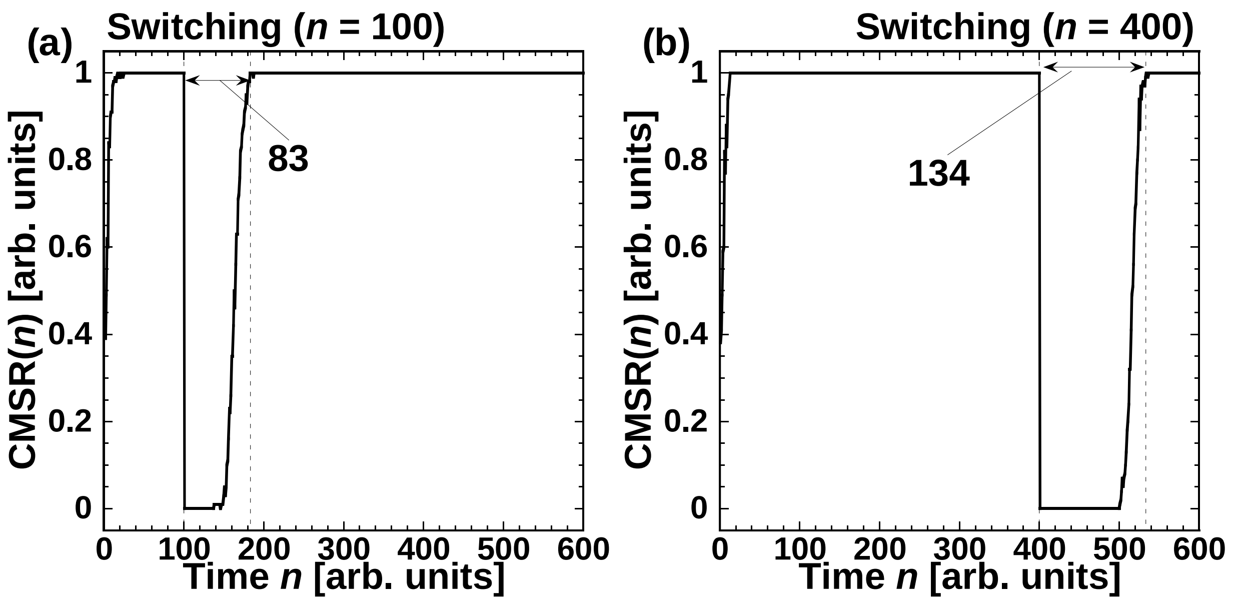

Figure S1. Temporal evolution of the correct model selection rate (CMSR). The switching from the Lorenz to Rössler

models occurs at (a) $n = 100$ and (b) $n = 400$.

The dependence of the transient time of the model selection on the switching time n is shown in Fig. S2. The horizontal axis represents the time n at which the switching occurs, and the vertical axis represents the transient time required for CMSR = 1. We discovered that the transient time increases for a greater time n at which the switching occurs. Therefore, the model selection succeeded in the cases at different switching times.

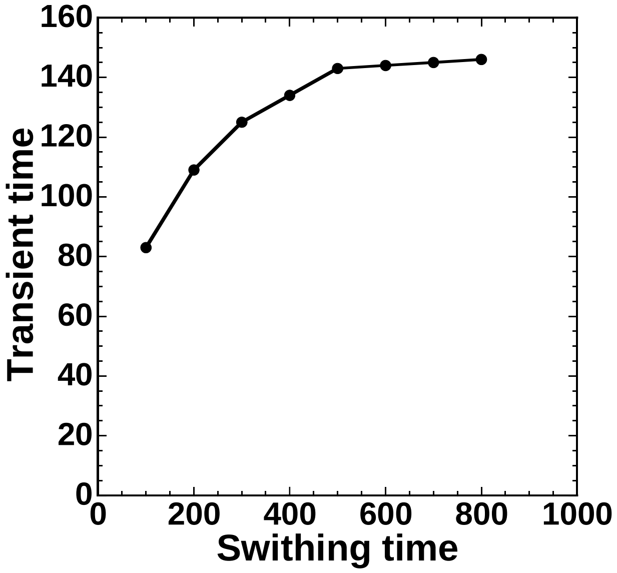

Figure S2. Dependence of the transient time required for CMSR = 1 on the switching time n.

The results of Fig. S2 can be explained by the temporal evolution of the threshold adjuster $TA(n)$. Figure S3 shows the temporal evolution of $TA(n)$ when the models are switched at $n = 100$ and $n = 400$ in Figs. 8(a) and S3(b), respectively. $TA(n)$increases monotonically before the model switching occurs. When the model is switched, the value of $TA(n)$ in Fig. S3(b) (at $n = 400$) is greater than the value in Fig. S3(a) (at $n = 100$). After the switching, the threshold adjuster starts decreasing. For Fig. S3(a), $TA(n)$ rapidly approaches zero and becomes a negative value because the value of the threshold adjuster is not large when the switching occurs. However, for Fig. S3(b) a longer transient time is required for the threshold adjuster to become a negative value because the value of the threshold adjuster is longer when the switching occurs. Therefore, a longer transient time is required for a longer switching time. This long transient time can be improved if the maximum and minimum values of the threshold adjuster are limited.

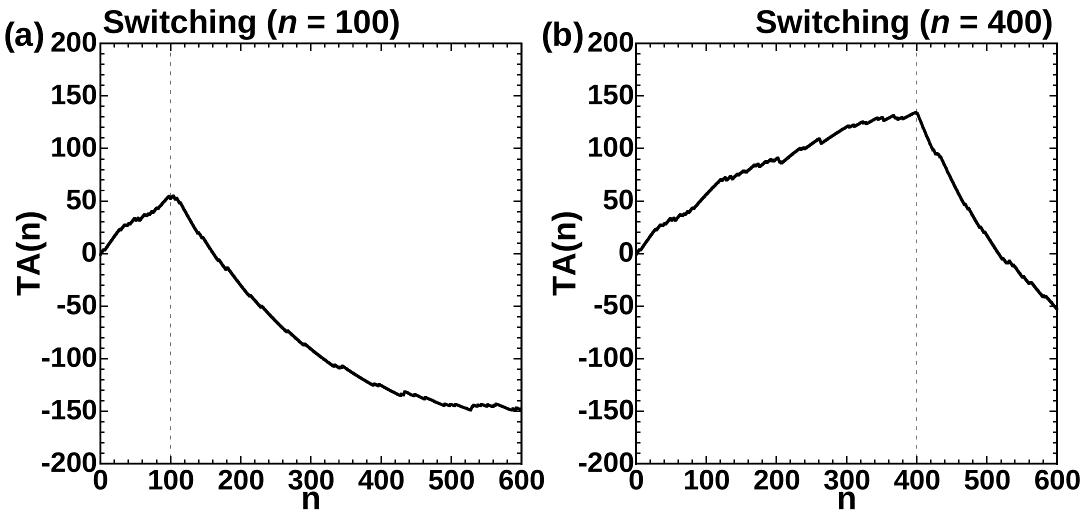

Figure S3. Temporal evolution of the threshold adjuster $TA(n)$. The switching from the Lorenz to Rössler models occurs at (a) $n = 100$ and (b) $n = 400$.
